# Abstracts and Selections

**Published:** 1908-07

**Authors:** 


### Abstracts and Selections.

### Medical Emergencies.—A Great Society for Coping With the Emergencies of Peace and War.

The National Volunteer Emergency Service, instituted in 1900, has recently been reorganized by the election of Dr. James Evelyn Pilcher, the distinguished editor of the *Military Surgeon,* as its Director General, and Dr. F. Elbert Davis, of New York, as its Adjutant General. Its work will be conducted along military lines, the details being worked out in three separate Corps, a First Aid Corps, a Public Health Corps, and a Medical Corps—the latter consisting of physicians, with rank of lieutenant to colonel, according to length of service, to whom are afforded special opportunities for emergency training. It includes among its personnel a large number of notable personages, and is rapidly extending its membership throughout the country. Full details regarding the service and its great work may be obtained by addressing Director General Pilcher at Carlisle, Pa.

### Privately Owned Medical Journals—An Objection Made to Them.

From time to time, the society and association organs have referred to "privately-owned" medical journals, as though the hyphenated adjective applied to them was incontestable evidence of illegitimacy. "Privately-owned" is written with the same distrust that is employed in penning the term "secret formula" or "advertised to the laity."

There are several American medical journals that are compelled to plead guilty to the charge of being "privately owned." Some are even shameless and brazen enough to admit that they contribute something to the material support of the "private owner." And what, pray, shall we do about it?

To be sure many of these shameless journals are owned by physicians—"*brother* practitioners" we have chosen to call them when the milk of human kindness is wet upon our lips. The charge, then, is that certain medical journals in being "privately owned" contribute to the support of certain physicians who find literary work not inconsistent with the honest practice of medicine. We have not arrived at that superlative ethical state where we see anything improper in that. But to be stigmatized as "privately owned" seems, in some quarters, to at least be placed under suspicion. Out of the placid hamlet of Berlin once came a publication which bore the ear-marks of being "privately owned" called *"Virchow's Archives,"* and we sincerely trust that the noble leader of medical thought who fathered it went to his grave without realizing that he was brazen and (at least) indelicate in "privately-owning" a journal.

The editors of the society and association organs, who pull in their immaculate skirts when they pass by the "privately-owned" in the broad road of life, are not recorded in the Book of Virtue as deriving no material compensation from their labor. In fact, some of the unregenerate contend that, virtuous though they may be, they have inscribed over their desks that scriptural passage which was so attractive to mother Mary Baker G. Eddy—"the servant is worthy of his hire." If deriving money from a journal constitutes the misconduct which causes the "privately-owned" journal to be viewed askance, we are unable to see how a fat salary abstracted from medical society coffers differs from that voluntarily paid by physicians by their own wish and volition. If there be a difference in the virtue of the incident, it would appear to us that the balance turns toward the latter method of compensation.

We are unable to appreciate the view taken of the "privately-owned" publication, perhaps because we are one of them. However, we can not resist the belief that, if, in the great scheme of organization, a coterie of medical men "representing the profession" should undertake the manufacture of surgical instruments as well as the publication of journals and directories, the "privately-made" obstetrical forceps would be too brazen and unnice an instrument to be used by a truly ethical medical man upon a modest and refined lady. And yet the "pulling-power" of the privately made forceps might be quite as strong as that of the orthodox instrument—as is, incidentally, the "pulling-power" and the educating power of the privately-owned journal as compared with that of the society organ.

Real ethics, brother practitioners of the old guard, is as clear and unmistakable as the noonday sun. Modern, hand-made ethics, like the love of God, "passeth all understanding."—*Chicago Clinic.*

---

AND NOW AS TO "PROPRIETARY MEDICINES."—We have, on more than one occasion, placed our views before the readers of this journal on this subject. The quotation in the communication of the Secretary, from Chapter 2, Article 1, Section 8, of "The Principles of Medical Ethics" is but a repetition of Article 1, Section 4, of

"The Duties of Physicians to Each Other, and to the Profession at Large," of the old ("Percival") "Code of Ethics," and was written long before the Science or Department of Pharmacy had advanced to the making of proprietaries. It is and has long been obsolete, and was not intended, as some "smart Alecs" would have us believe, to place a ban and a bar to this decided advance. There are "proprietaries and proprietaries." Some have become as well-nigh "standard" as any drug or medicament can well be. Others have proven useless, and such are already, or soon will be discarded, and that without any pronunciamento from any 7x8 council. Among our teachers in medicine we have seen three elected to preside over the American Medical Association, and we have ever held closely to their teachings. Becoming a member of the Association more than thirty years ago, we have ever held allegiance to it, and have added our humble efforts to its progress in so far as we were able, and in doing so, we have never surrendered our inalienable right to think for ourself. The proprietaries represented in the pages of this journal have all had place in the pages of the *Journal of the American Medical Association*. If such men in charge of the *Journal A. M. A.* as the "Father of the Association," Drs. J. C. Culbertson, Jno. H. Hollister and Jno. B. Hamilton could sanction their appearance there, I feel that I could far better "follow their lead" than submit to the dictation of any council whatever, regardless of its selection or the fine Italian hand holding the reins.—*Southern Practitioner, Nashville.*

---

I knowed a feller onc't that had
The yeller janders mighty bad—
And each and ev'ry friend he'd meet
Would stop and give him some receet
Fer cuorin' of 'em. But he'd say
He kindo' thought they'd go away
Without no medicin', and boast
That he'd git well without one doste.

He kep' a-yellerin' on—and they
Perdictin' that he'd die some day
Before he knowed it. Tuk his bed,
The feller did, and lost his head
And wundered in his mind a spell—
Then rallied, and, at last, got well;
But ev'ry friend that said he'd die
Went back on him eternally! —*Riley.*

### Congress Sits Up and Takes Notice.

The Senate Committee on Public Health and National Quarantine, through Senator Daniel, its chairman, has made a favorable report on a bill that it is expected if enacted will afford better protection of the public health and provide for the investigation of disease, the diffusion of the information thus obtained and the co-operation of the Public Health and the Marine Hospital Service with State and municipal health authorities for the suppression of diseases.

It is the belief of the committee that the bill in question will make the countrys public health service much more efficient and will go far to check the spread of various diseases. One method of doing this is through special investigations into the prevalence of tuberculosis, typhoid fever, rabies and other diseases.

It is proposed to bring about special study of the condition favoring their propagation and spread and the methods necessary for their suppression. It is considered of much importance that knowledge of the geographical distribution of these diseases should be extended, that their modes of propagation should be determined, and that the people should be informed of the measures necessary to their prevention.

That more thorough protection of the public health is economically important is shown by the fact, the committee says, that the losses caused by tuberculosis alone cost the county, on a conservative estimate, $300,000,000 annually. It is conservatively estimated that in the United States alone one hundred and fifty thousand persons die annually of tuberculosis, a preventable and curable disease. That it is curable is proven by the fact that in the fiscal year 1907, 101 recovered cases were discharged from the United States Hospital Sanitarium at Fort Stanton, N. M.

In the single matter of tuberculosis alone, the committee takes the view that much can be done by giving to the Public Health and Marine Hospital Service fuller authority and fuller means for its study and for circulation of the information that is obtained. Tuberculosis is favorably influenced by climatic treatment, but in the absence of definite knowledge regarding the factors contributing thereto, much hardship and even death has followed the indiscriminate migrations of patients from one section of the country to others in search of health.

As a result of these migrations deplorable conditions have arisen in certain sections and reputed health resorts have become insanitary resorts. The committee says, too, that the transportation of these cases by interstate carriers also constitutes a real danger and means must soon be provided to insure the greater safety of travelers with whom they come in contact.

One part of the plan, as embodied in the bill, is the distribution widely to the public of plain, simple, sanitary bulletins dealing with diseases, their nature, how to prevent them, how to treat them, and the like. In other words, it is proposed to send the whole country to school in all subjects relating to disease, hygiene and sanitation.—*New York Herald.*

---

### Army Medical Department Examinations, 1908.

The Act of April 23, 1908, reorganizing the medical corps of the army, gives an increase in that corps of six colonels, twelve lieutenant colonels, forty-five majors, and sixty captains or first lieutenants, and establishes a medical reserve corps as an adjunct to the medical corps. Under this recent act, the lieutenants of the medical corps are promoted to captain after three years' service instead of five, and the increase in the higher grades insures promotion at a reasonable rate all through an officer's military career. Furthermore, applicants who are found qualified in the preliminary examinations are appointed first lieutenants of the medical reserve corps and ordered to the Army Medical School in Washington, D. C., for eight months' instruction.

### THE MEDICAL CORPS.

Preliminary examination for appointment in the medical corps will be held on August 3, 1908, and formal applications should be in possession of the War Department prior to July 1st. The applicant must be a citizen of the United States, between 22 and 30 years of age, a graduate of a medical school legally authorized to confer the degree of doctor of medicine, of good moral character and habits, and must have had at least one year's hospital training or its equivalent in practice. The examination will be held concurrently throughout the country at points where boards can conveniently be assembled, and due consideration will be given to the localities from which applications are received, in order to lessen the traveling expenses of applicants as much as possible.

The examination in subjects of general preliminary education may be omitted in the case of applicants holding diplomas from reputable literary or scientific colleges, normal schools or high schools, or graduates of medical schools which require an entrance examination satisfactory to the faculty of the Army Medical School.

The large number of vacancies created in the medical corps by recent legislation makes it certain that *all* successful candidates will be recommended for a commission for several years to come.

THE MEDICAL RESERVE CORPS.

It is desired to obtain and maintain a list of qualified medical men all over the country who are willing to serve as medical officers in time of emergency, and to such men the President is authorized to issue commissions as first lieutenants, medical reserve corps. It is recognized that it will be necessary to place only a limited number of these officers on the active list in time of peace, and it is hoped that young medical men throughout the country and medical officers of the militia of the various States may be sufficiently interested to secure positions on the medical reserve corps list.

An applicant must be between 22 and 45 years of age, a citizen of the United States, a graduate of a reputable medical school legally authorized to confer the degree of doctor of medicine, and must have qualified to practice medicine in the State in which he resides. Examinations will be held in the near future and will embrace the practical medical subjects.

Full information concerning the medical corps and the medical reserve corps may be procured upon application to the Surgeon General, U. S. Army, Washington, D. C.

---

### The Edward N. Gibbs Memorial Prize of One Thousand Dollars.

Subject—The Etiology, Pathology and Treatment of the Diseases of the Kidney.

The New York Academy of Medicine announces that the sum of $1000 will be awarded to the author of the best essay in competition for the above mentioned prize.

The subject of the essay, as stated, shall be, "The Etiology, Pathology and Treatment of the Diseases of the Kidney."

Essays must be presented on or before October 1, 1909.

The three subjects mentioned in the title as above given need not be treated with uniform fullness, but new discovery or fruitful research will be considered the standard of merit.

Each essay must be in English, typewritten, designated by a motto, or device, and accompanied by a sealed envelope, bearing the same motto, or device, which shall contain the name and address of the author.

No envelope will be opened except that which accompanied the successful essay.

The Academy reserves the right, according to the direction of the donors, not to award the prize if no essay shall be deemed worthy of it.

The Academy will return the unsuccessful essays, if claimed by their respective authors, or by authorized agents, within six months.

An essay must show originality in order to obtain the prize.

The competition is open to the members of the regular medical profession of the United States.

The original of the successful essay shall be the property of the Academy, and, according to the deed of gift, will be published in its Transactions.

The essays shall be transmitted to the Committee of the New

York Academy of Medicine on the Edward N. Gibbs Memorial Prize.

JOHN A. WYETH, President.

JOHN H. HUDDLESTON, Recording Secretary.

The New York Academy of Medicine.

New York, June 1, 1908.

---

### The Danger and Prevention of Tetanus From Fourth of July Wounds.

BY JOHN F. ANDERSON,
Past Assistant Surgeon, Public Health and Marine Hospital Service.

For the past five years the *Journal of the American Medical Association* has, before each Fourth of July, pointed out in its editorial columns, the special danger of tetanus subsequent to wounds incurred in the use of blank cartridge pistols, toy cannons, firecrackers, and other noise-producing devices. Some time after the Fourth of July, when the full effects can be counted in deaths from tetanus, loss of limbs, burns, loss of eyes and eyesight, etc., it has published as complete a list as possible of the cost to the nation in lives and injuries.

The following table, taken from the *Journal*, shows the number of cases of tetanus following the celebration of the Fourth for the past five years:

| Year. | Blank cartridge. | Giant cracker. | Cannon. | Firearm. | Powder, etc. | Total cases. | Total deaths |
|---|---|---|---|---|---|---|---|
| 1903 | 363 | 17 | 5 | 3 | 27 | 415 | 406 |
| 1904 | 74 | 18 | 5 | 1 | 7 | 105 | 91 |
| 1905 | 65 | 17 | 4 | 5 | 13 | 104 | 87 |
| 1906 | 54 | 17 | 1 | 7 | 10 | 89 | 75 |
| 1907 | 52 | 8 | 6 | 4 | 3 | 73 | 73 |
| Total | 608 | 77 | 21 | 20 | 60 | 786 | 722 |

The great decrease in the number of cases from 415 in 1903 to 73 in 1907 must be attributed in large measure to the campaign led by the *Journal* and ably seconded by the lay press for the more thorough and careful treatment of Fourth of July wounds, and especially the use of tetanus antitoxin as a prophylactic. From the table it will be seen that there have been 786 cases of tetanus, with 721 deaths, a mortality of 92 per cent of those attacked. Six hundred and eight, or 80 per cent, of these followed blank cartridge wounds. The number of cases of lockjaw following blank catridge wounds has steadily decreased since 1903 from 363 to 52

in 1907, while the cases following other wounds have remained fairly constant. This would seem to indicate that the public is realizing the great danger from blank cartridges.

*Distribution of the Tetanus Bacillus.*—The bacillus of tetanus, or its spore form, is found in earth, especially garden earth, stable manure, the dust of the streets, stables, and human habitations. The feces of healthy animals, such as the horse, cow, dog, and even man, may contain tetanus spores.

Verneuil considers the intestinal tract of the horse the normal habitat of the organism and, as the dust of the streets is largely horse manure, it is easy to see why wounds contaminated in any way by this dust are so liable to be followed by tetanus. Mixed infections are especially dangerous, as other organisms, especially the pus cocci, assist in the production of anaerobic conditions and prevent the leucocytes from destroying the tetanus spores, as may happen when they are inoculated into animals in pure culture.

The tetanus spore is very resistant to heat, as shown by the fact that Theobald Smith has found some strains that resist boiling for seventy minutes. The question of the presence or absence of tetanus bacilli in blank cartridges is discussed pro and con in an article in the *Journal of the American Medical Association** and the weight of evidence is certainly in favor of the view that blank cartridges do not contain tetanus bacilli except as a rare contamination when they have been exposed to dirt. There is ample evidence that tetanus spores are present in the street dirt and thus get on the skin and clothing of the injured, and are carried into the wounds by the discharge of the pistol.

*Pathology of Tetanus.*—When tetanus spores are introduced under the skin they at once germinate, if oxygen is excluded, and begin to elaborate tetanus toxin. This toxin consists of at least two poisons—*tetano-lysin* and *tetano-spasmin;* the latter is the poison that causes lockjaw.

The motor nerves have a special affinity for tetanus toxin. As soon as the toxin is produced the adjacent motor nerve-endings at once begin to take it up and transport it to the cord. The lymphatics also absorb much of it and in a short time it appears in the blood, which carries it to all parts of the body, where it is absorbed by the motor nerve-endings which are bathed in the toxin-laden fluid. The nerves supplying the jaws, especially the masseteric, seem to have an exaggerated affinity for the poison, which explains the early trismus. The symptoms are usually earlier and

*The Fourth of July, 1903, Casualty List. Jour. Am. Med. Assn., Vol. 41, p. 547.

more marked in the limb in which the wound is present, due to the fact that the toxin index is higher there than in other parts of the body. The evidence is decidedly in favor of the view that the toxin produces its effect solely by its absorption by the nerves whose axis-cylinder substance has a specific affinity for the poison; they transport it to the cord and brain through the axis-cylinder substance.

The affinity of the nerves for the toxin and its subsequent binding by them explains why tetanus antitoxin is of very little value after the symptoms have appeared. Antitoxin can, however, neutralize any new toxin that may be formed in cases in which the focus has not been removed.

The incubation period may be from four or five days to several weeks. If the incubation period is four or five days, death frequently occurs in twenty-four to seventy-two hours. The more prolonged the incubation period is over ten days the better chance there is for recovery. The few cases that do recover are almost invariably those with an incubation period of ten days or more. The mortality of those attacked is from 90 to 95 per cent.

*Treatment of Fourth of July Wounds.*—Physicians often treat blank cartridge and other Fourth of July injuries, unless of a serious character, as trivial, contenting themselves with picking out the plainly seen pieces of wad, powder, etc., and applying a dressing. In a few days the patient returns with symptoms of tetanus and then, when it is too late, antitoxin is given, the wound is thoroughly cleaned out, and perhaps a piece of wad or clothing found in it.

It should be an invariable procedure that all Fourth of July wounds be laid fully open under local or, preferably, general anesthesia and all foreign material and necrotic or badly injured tissue removed, as the presence of blood clots and necrotic tissue favor anaerobic conditions, which are essential for the development of the tetanus organism. The use of a constrictor, such as the Esmarch bandage, to prevent hemorrhage during the operation if the wound is on a limb is of much assistance. After the wound has been thoroughly cleaned out it should be swabbed out with strong carbolic acid, at least 25 per cent, followed by a washing with 95 per cent alcohol to prevent further action of the acid. Some surgeons use peroxide of hydrogen instead of carbolic acid. On account of the action of formalin* on tetanus toxin I think swabbing out the wound with pure formalin after the thorough surgical cleaning out would be an excellent procedure; the pain from the use of formalin soon passes off, and the necrosis caused by it is slight. I hope it will be given a trial by surgeons in Fourth of July wounds.

After cauterization, by whatever method used, the wound should be thoroughly washed out with a 1-1000 or 1-2000 solution of bichloride of mercury and packed with gauze soaked in a saturated solution of salicylic or boric acid and a large wet dressing of the same solution applied. In no case should the wound be closed, but it should be allowed to heal by granulation. The dressing and packing should be renewed every day.

*Tetanus Antitoxin.*—Immediately after the wound has been dressed as above at least 1500 units of tetanus antitoxin should be given. This may be given in the loose tissue of the abdomen, in

---

*Anderson, John F.: The antiseptic and germicidal properties of solutions of formaldehyde and their action upon Toxins. Hygienic Laboratory Bulletin No. 39, 1907.

the buttocks, between the scapulae, or in any convenient part of the body. It is best to administer the antitoxin while the patient is in the recumbent position. The administration of antitoxin is the most important part of the whole treatment.

The following table, taken from an article by Scherck,* of St. Louis, shows in a most convincing way the great value of tetanus antitoxin as a prophylactic in Fourth of July wounds:

*Cases of Fourth of July Wounds Treated in St. Louis City Dispensaries.*

| Year. | Cases of wounds treated. | Antitetanic serum used. | Deaths from tetanus. |
|---|---|---|---|
| 1903 ............................ | 56 | None. | 16 |
| 1904 ............................ | 37 | Yes. | .... |
| 1905 ............................ | 84 | Yes. | .... |
| 1906 ............................ | 170 | Yes. | .... |

It will be seen that in 1903, when no tetanus antitoxin was used, 16 or about 30 per cent of the 56 cases of Fourth of July injuries treated died from tetanus; in 1906, 170 cases of similar injuries were treated, antitoxin being used, *without a single death* from tetanus.

*Résumé of Treatment.*—The procedure reported in the same article by Scherck for the surgical handling of Fourth of July injuries seems to be so satisfactory that I quote it verbatim:

1. To incise freely every wound.
2. Carefully and thoroughly to remove from the wound every particle of foreign matter.
3. To cauterize the wound thoroughly with 25 per cent carbolic acid.
4. To apply loosely a wet pack of 2.5 per cent carbolic acid.
5. To give a full dose of antitetanic serum.

The patient was required to return every day for observation and dressing of the wound.

If the various municipalities would pass laws restricting the sale of firearms and ammunition to minors, absolutely prohibit the sale of blank cartridges, toy cannons, and giant fire crackers, and through the lay press educate the people to the great danger of Fourth of July wounds and the importance of thorough surgical treatment and the prophylactic use of *tetanus antitoxin,* many valuable lives would be saved to the nation.

*Federal Control of Tetanus Antitoxin.*—An Act of Congress

---

*Scherck, H. J.: Antitetanic serum in Fourth of July injuries. Jour. Am. Med. Assn., Vol. 47, p. 500.

approved July 1, 1902, provides that establishments engaged in the manufacture and interstate sale of viruses, serums, toxins, and analogous products shall be licensed by the Secretary of the Treasury upon the recommendation of the Surgeon General of the United States Public Health and Marine Hospital Service, after inspection by an officer of that service of the stables, laboratories and methods used and an examination of their products in the Hygienic Laboratory. Samples of the products put on sale by licensed manufacturers are bought at frequent intervals on the open market and examined in the Hygienic Laboratory for purity and potency. Up to October 25, 1907, there was no official standard for testing tetanus antitoxin in the United States; each manufacturer had his own arbitrary method of determining the potency of his products. On that date, however, an American unit was officially promulgated by the Secretary of the Treasury on the recommendation of the Surgeon General of the Public Health and Marine Hospital Service.

This American standard is the result of several years' work in the Hygienic Laboratory and commends itself on account of its simplicity, directness and accuracy. The unit is defined as "ten times the least quantity of antitetanic serum necessary to save the life of a 350-gram guinea pig for ninety-six hours against the official test dose of a standard test toxin furnished by the Hygienic Laboratory of the Public Health and Marine Hospital Service." The Society of American Bacteriologists in December, 1906, adopted a resolution in regard to the standardization of tetanus antitoxin and decided that the minimal immunizing dose for a case of possible tetanus infection should be 1500 units.

The great need of an official standard for tetanus antitoxin was shown by the examination of samples of serum sold in the United States. It was found, before the adoption of the American unit, that the serums varied extravagantly in unit strength claimed and the actual number of units they contained. Since the unit has been established there has been uniformity in the product and a decided increase in potency. When a physician now buys a package of tetanus antitoxin in the United States he can rest assured that it contains a potent product and the number of units indicated on the label.—*From Public Health Reports* for June, 1908, U. S. Public Health and Marine Hospital Service.

